# Association of Dietary Vitamin A and β-Carotene Intake with the Risk of Lung Cancer: A Meta-Analysis of 19 Publications

**DOI:** 10.3390/nu7115463

**Published:** 2015-11-11

**Authors:** Na Yu, Xinming Su, Zanfeng Wang, Bing Dai, Jian Kang

**Affiliations:** Department of Respiratory Medicine, The First Hospital of China Medical University, No.155 Nanjing North Street, He-ping District, Shenyang 110001, Liaoning, China; xinmingsu123@yeah.net (X.S.); zanfengwang123@yeah.net (Z.W.); bingdai123@yeah.net (B.D.); kangjian58@163.com (J.K.)

**Keywords:** vitamin A, β-carotene, lung cancer, meta-analysis

## Abstract

Whether dietary β-carotene and vitamin A intake protect against lung cancer risk is not clear. Therefore, we performed this meta-analysis to investigate the association between them. The related articles were searched using the databases PubMed and the Web of Knowledge up to May 2015. We used the random-effect model to estimate the relative risk (RR) and their 95% CI. Small-study effect was assessed using Egger’s test. In total, 19 studies comprising 10,261 lung cancer cases met the inclusion criteria. The pooled RR and their 95% CI was 0.855 (0.739–0.989) for higher category of dietary vitamin A intake and lung cancer risk, especially among Asian populations and in the cohort studies. Evidence from 18 studies suggested that higher category of dietary β-carotene intake could reduce lung cancer risk (0.768 (0.675–0.874)).The associations were also significant in American and Asian populations. In conclusions, higher category of dietary β-carotene and vitamin A intakes could reduce the risk of lung cancer. However, the dose-response analysis was not performed due to the limited data in each individual study. Due to this limitation, further studies with detailed dose, cases and person-years for β-carotene and vitamin A of each category are wanted to assess this dose-response association.

## 1. Introduction

Lung cancer is a leading cause of cancer mortality worldwide [1] and the overall survival rate is still extremely poor [2]. Lung cancer is a fatal disease with a complex carcinogenesis mechanism. In addition, the incidence rate was 62.6/100,000 per year while the death rate was 50.6/100,000 per year worldwide in 2011 [3]. Therefore, to prevent lung cancer is an important matter in current society.

Two recent meta-analyses had been performed to evaluate the relationship between vitamin C [4] and vitamin E [5] and lung cancer risk. The result indicated that lung cancer risk would decrease by 7% with every 100 mg/day increased vitamin C intake [4]. In addition, the lung cancer could decrease by 14.2% for higher dietary vitamin E intake [5]. Dietary antioxidants have been shown in laboratory studies to impede the growth of cancer cells in general [6,7]. Vitamin A and β-carotene may be involved antioxidant activity, induction of detoxifying enzymes, and inhibition of cellular proliferation. This may be an important role in lung cancer prevention [8]. Furthermore, vitamin A and β-carotene are involved in methylation of DNA and DNA damage [8]. The World Cancer Research Fund and the American Institute for Cancer Research (WCRF/AICR) [9] in 2007 had reported that dietary vitamin A and β-carotene intake from both cohort and case-control studies are associated with decrease lung cancer risk. However, supplements of β-carotene could increase the risk of lung cancer. Since then, two studies were published to further explore the relationship between dietary vitamin A and β-carotene intake and lung cancer risk [10,11]. As the associations from the published studies were not consistent [10,11,12,13], we conducted an update meta-analysis to further evaluate the evidence from observational studies on vitamin A and β-carotene intake and lung cancer risk.

## 2. Methods

### 2.1. Search Strategy

A comprehensive literature search was performed from 1990 to May 2015 on PubMed and the Web of Knowledge. The following keywords were used in the search: “vitamin A” or “β-carotene” combined with “lung cancer” or “lung carcinoma” without any restrictions. Moreover, the references of the included studies were also reviewed and identify additional studies which were not captured by our computer searches.

### 2.2. Inclusion Criteria

The studies were considered if they fulfilled the following inclusion criteria: (1) they were observational studies; (2) reported dietary vitamin A or retinol or β-carotene intake; (3) the ending outcome was lung cancer; (4) multivariate-adjusted relative risks (RR) or odds ratio (OR) with 95% confidence intervals (CI) were provided. We excluded the studies if they were: (1) abstracts and (2) overlapped publications.

### 2.3. Data Extraction

The following data of included studies was extracted: first author’s last name, study design, publication years, country where the study was performed, number of cases, sample size, sex, the histological type of lung cancer, variables adjusted for in the analysis, the category of RR and their 95% CI for dietary vitamin A and β-carotene intake, respectively. We only extracted the results of dietary vitamin A and β-carotene intake if the total intake were available. From each study, the multivariable adjustment RR and 95% CI were extracted. Otherwise, the crude RR was useful.

### 2.4. Statistical Analysis

The RR and 95% CI was pooled, which considers both within-study and between-study variation, to assess the association between vitamin A or β-carotene intake and lung cancer risk using the random-effects model [14]. The between-study heterogeneity was assessed using *I*^2^, and *I*^2^ values of 0%–25%, 25%–50%, 50%–75% and >75% represent no, low, moderate and high heterogeneity [15], respectively. Subgroup analysis and meta-regression were performed to explore the potential heterogeneity [16]. An Egger regression asymmetry test was used to evaluate the small-study effect [17]. Sensitivity analysis [18] was conducted to describe whether the results could be affected once one study removed at a time. All analyses were performed using STATA version 12.0 (Stata Corporation, College Station, TX, USA). Two-sided with *p* < 0.05 was considered statistically significant.

## 3. Results

### 3.1. Characteristics of Included Studies

**Figure 1:**
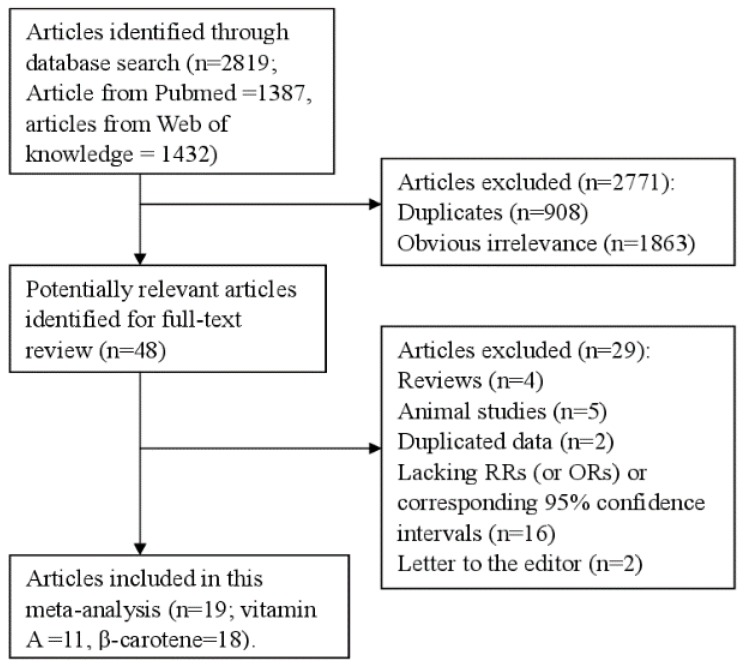
The flow diagram of screened, excluded, and analyzed publications.

Figure 1 showed the detailed steps of the literature search. We identified 2819 relevant articles from our databases, and 48 articles left after reviewing the title/abstract. Four review articles, five articles reporting the animal studies, two duplicated articles, 16 articles lacking the RR or 95% CI, and two letter to the editor articles were further excluded after full-text review. Hence, 19 articles [10,11,12,13,19,20,21,22,23,24,25,26,27,28,29,30,31,32,33] involving 10,261 lung cancer cases were included. Ten studies were conducted from United States, two from the Netherlands, three from China, two from Canada, one from Finland and one from Uruguay. Table 1 showed the detailed characteristics of the included studies.

### 3.2. Vitamin A and Lung Cancer

**Figure 2:**
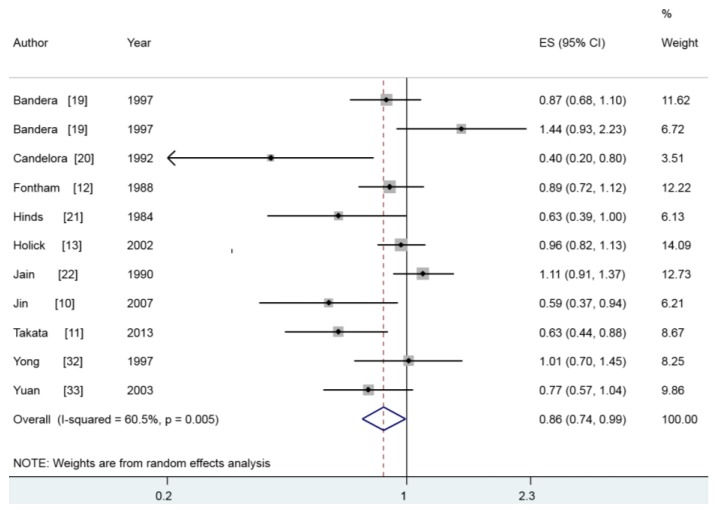
The multivariate-adjusted risk of lung cancer for the highest *versus* lowest categories of vitamin A intake.

Ten articles [10,11,12,13,19,20,21,22,32,33] with 11 studies including 6139 lung cancer cases reported the association between dietary and lung cancer risk. Inverse association was reported in four studies, while seven studies did not find positive results. In our study, we concluded that dietary vitamin A intake could reduce lung cancer risk (summary RR = 0.855, 95% CI = 0.739–0.989, *I*^2^ = 60.5%) (Figure 2). In the subgroup analysis of study design, the combined RR with their 95% CI were 0.869 (0.758–0.980) and 0.754 (0.560–1.016) for prospective studies and case-control studies, respectively. When we stratified studies by geographic locations, we found an inverse association between dietary vitamin A intake and lung cancer risk among Asian populations (summary RR = 0.682, 95% CI = 0.556–0.837), but not in the American populations. The subgroup analysis by sex was also performed, and the association was significant only in males (summary RR = 0.697, 95% CI = 0.553–0.879). Table 2 showed the detailed results.

**Table 1:** Characteristics of studies on dietary vitamin A and β-carotene intake with the risk of lung cancer.

Study, Year	Country	Study Design	Participants (Cases)	Age (Years)	Categories	RR (95% CI) for Each Category	Adjustment for Covariates
Bandera *et al*., 1997 [19]	United States	Prospective	48,000 (525)	40–80	Vitamin A Males Tertiles 1 Tertiles 2 Tertiles 3 Females Tertiles 1 Tertiles 2 Tertiles 3	Vitamin A Males 1 0.82 (0.64–1.05) 0.87(0.68–1.10) Females 1 1.19(0.75–1.88) 1.44(0.93–2.23)	Adjusted for age, education, cigarettes/day, years smoking, and total energy intake (except calories) based on Cox Proportional Hazards Model.
Candelora *et al.*, 1992 [20]	United States	Case-control	387 (124)	Case: 71.9 Control: 69.8	Vitamin A Quartile 1 Quartile 2 Quartile 3 Quartile 4 β-carotene Quartile 1 Quartile 2 Quartile 3 Quartile 4	Vitamin A 1 0.60(0.30–1.20) 0.60 (0.30–1.20) 0.40 (0.20–0.80) β-carotene 1 0.50 (0.30–1.00) 0.50 (0.30–0.90) 0.40 (0.20–0.80)	Adjusted for age, education (≤8 and >8 grades), and total calories.
Fontham *et al.*, 1988 [12]	United States	Case-control	2527 (1253)	<40–≥70	Vitamin A Low Moderate High β-carotene Low Moderate High	Vitamin A 1 0.85 (0.68–1.06) 0.89 (0.72–1.12) β-carotene 1 0.96 (0.76–1.20) 0.88 (0.70–1.11)	Adjusted in logistic regression model for age, race, sex, and pack years of cigarette use.
Hinds *et al.*, 1984 [21]	United States	Case-control	991 (364)	≥30	Vitamin A (IU) 0–51,799 51,800–78,099 78,100–115,199 115,200 +	Vitamin A 1 0.88 (0.49–1.26) 1.06 (0.58–1.54) 0.63 (0.39–1.00)	Adjustment by multiple logistic regression for age, ethnicity, cholesterol intake, occupational status, vitamin A intake, pack-years of cigarette smoking, and sex where appropriate.
Holick *et al.*, 2002 [13]	Finland	Prospective	27,084 (1644)	50–69	Vitamin A (μg/day) <717 717–1044 1045–1481 1482–2138 >2138 β-carotene (μg/day) <977 977–1440 1441–2029 2030–3015 >3015	Vitamin A 1 0.97(0.83–1.14) 1.02(0.87–1.20) 1.03(0.88–1.21) 0.96(0.82–1.13) β-carotene 1 0.92(0.79–1.06) 0.90(0.78–1.04) 0.79(0.68–0.92) 0.92 (0.79–1.07)	Adjusted for age, years smoked cigarettes per day, intervention (α-tocopherol and β-carotene supplement), supplement use (β-carotene and vitamin A), energy intake, cholesterol, and fat.
Jain *et al.*, 1990 [22]	Canada	Case-control	1611 (839)	20–75	Vitamin A Highest *vs.* Lowest β-carotene Highest *vs.* Lowest	Vitamin A 1.11 (0.91–1.37) β-carotene 1.00 (0.79–1.27)	Adjusted for cumulative cigarette smoking
Jin *et al.*, 2007 [10]	China	Case-control	903 (301)	≤80	Vitamin A (RE/day) ≤947 947–1742 1742–3630 ≥3630 β-carotene (μg/day) ≤3734 3735–7440 7440–15,363 ≥15,363	Vitamin A 1 0.78 (0.51–1.18) 0.62 (0.41–0.95) 0.59 (0.37–0.94) β-carotene 1 0.74 (0.49–1.12) 0.69 (0.45–1.06) 0.52 (0.32–0.83)	Adjusted for pack-years of cigarette smoking, occupational exposure, passive smoking exposure from mother and friends, medical insurance status and education levels.
Le Marchand *et al.*, 1989 [23]	United States	Case-control	1197 (332)	30–85	β-carotene Males Quartile 1 Quartile 2 Quartile 3 Quartile 4 Females Quartile 1 Quartile 2 Quartile 3 Quartile 4	β-carotene Males 1 1.25 (0.76–1.74) 0.81 (0.52–1.10) 0.63 (0.36–1.11) Females 1 0.81 (0.51–1.11) 0.62 (0.31–0.93) 0.39 (0.16–1.00)	Adjusted for age, ethnicity, smoking status, pack-years of cigarette smoking, cholesterol intake (for males only), and intakes of other nutrients in the table.
Neuhouser *et al.*, 2003 [24]	United States	Prospective	14,120 (742)	Case: 60.4 Control: 57.6	β-carotene (μg/day) ≤1156 1157–1714 1715–2331 2332–3428 ≥3429	β-carotene 1 0.90 (0.63–1.28) 0.92 (0.65–1.30) 1.03 (0.73–1.45) 0.95 (0.67–1.36)	Adjusted for sex, age, smoking status, total pack-years of smoking, asbestos exposure, race/ethnicity, and enrollment center.
Ocke *et al.*, 1997 [25]	Netherlands	Prospective	561 (54)	Case: 59.3 Control: 59.5	β-carotene (mg) <1.07 1.07–1.31 >1.31	β-carotene 1 0.61 (0.21–1.04) 0.74 (0.41–1.35)	Adjusted for age, pack-years of cigarettes, and energy intake,
Rohan *et al.*, 2002 [26]	Canada	Prospective	5516 (155)	40–59	β-carotene Quartile 1 Quartile 2 Quartile 3 Quartile 4	β-carotene 1 1.78 (1.04–3.05) 1.83 (1.03–3.24) 1.40 (0.76–2.59)	Adjusted for age, study allocation, study center, cigarette smoking, vitamin C intake, folate intake, dietary fiber intake, and energy intake.
Speizer *et al.*, 1999 [27]	United States	Prospective	121,700 (593)	30–55	β-carotene Q5 *vs.*Q1	β-carotene 0.80 (0.60–1.11)	Age, total energy intake, smoking (past and current amount in 1980; 1±4, 5±14, 15±24, 25±34, 35±44, 45+) and age of starting to smoke.
Stefani *et al.*, 1999 [28]	Uruguay	Case-control	981 (541)	30–89	β-carotene (μg/day) <1938 1939–3330 3331–5862 ≥5863	β-carotene 1 0.83 (0.56–1.22) 0.61 (0.42–0.89) 0.42 (0.28–0.63)	Adjusted for age, residence, urban/rural status, education, family history of a lung cancer in 1st-degree relative, body mass index, tobacco smoking (pack-yr), and total energy and total fat intakes, IQR, interquartile range.
Steinmetz *et al.*, 1993 [29]	United States	Prospective	41,837 (179)	55–69	β-carotene Quartile 1 Quartile 2 Quartile 3 Quartile 4	β-carotene 1 0.76 (0.47–1.25) 0.67 (0.39–1.14) 0.81 (0.48–1.38)	Adjusted by inclusion of continuous variables for age, energy intake, and pack-years of smoking in multivariate logistic regression models.
Takata *et al.*, 2013 [11]	China	Prospective	61,491 (359)	40–74	Vitamin A (μg/day) 359.4 549.8 729.2 1046.1 β-carotene (μg/day) 1449.8 2045.8 3346.9 5025.5	Vitamin A 1 0.86 (0.65–1.13) 0.85 (0.64–1.14) 0.63 (0.44–0.88) β-carotene 1 0.83 (0.63–1.09) 0.82 (0.62–1.10) 0.64 (0.46–0.88)	Adjusted for age, years of smoking, the number of cigarettes smoked per day, current smoking status, total caloric intake, education, BMI category, ever consumption of tea, history of chronic bronchitis, and family history of lung cancer among first-degree relatives.
Voorrips *et al.*, 2000 [30]	Netherlands	Prospective	58,279 (939)	55–69	β-carotene Quartile 1 Quartile 2 Quartile 3 Quartile 4 Quartile 5	β-carotene 1 0.83 (0.60–1.14) 1.00 (0.72–1.39) 1.14 (0.80–1.62) 1.11 (0.76–1.60)	Adjusted for current smoking, years of smoking cigarettes, number of cigarettes per day, highest educational level, family history of lung cancer, and age.
Wright *et al.*, 2003 [31]	United States	Case-control	1211 (587)	35–84	β-carotene (μg/day) <823.58 823.58–1145.95 1145.96–1526.06 1526.07–2323.54 >2323.54	β-carotene 1 0.71 (0.49–1.00) 0.60 (0.41–0.87) 0.71 (0.48–1.10) 0.58 (0.39–0.86)	Adjusted for age, total calorie intake, pack-years of smoking, and education.
Yong *et al.*, 1997 [32]	United States	Prospective	1068 (248)	25–74	Vitamin A (IU) 1 2 3 4 β-carotene (IU) 1 2 3 4	Vitamin A 1 0.98 (0.68–1.39) 0.98 (0.69–1.40) 1.01 (0.70–1.45) β-carotene 1 0.66 (0.46–0.94) 0.78 (0.56–1.10) 0.74 (0.52–1.06)	Adjusted for sex race, educational attainment, nonrecreabonal activity level, body mass index, family history, smoking status/pack-years of smoking, total calorie intake, and alcohol intake.
Yuan *et al.*, 2003 [33]	China	Prospective	63,257 (482)	45–74	Vitamin A Quartile 1 Quartile 2 Quartile 3 Quartile 4 Quartile 5 β-carotene Quartile 1 Quartile 2 Quartile 3 Quartile 4 Quartile 5	Vitamin A 1 0.71 (0.54–0.92) 0.75 (0.58–0.99) 0.99 (0.76–1.28) 0.77 (0.57–1.04) β-carotene 1 0.77 (0.59–1.00) 0.81 (0.62–1.06) 0.98 (0.75–1.28) 0.85 (0.63–1.14)	Adjusted for age at baseline, sex, dialect group, year of interview, level of education, and BMI, number of cigarettes smoked per day, number of years of smoking, and number of years since quitting smoking for former smokers.

Abbreviations: BMI, body mass index; CI, confidence interval; RR, relative risk.

**Table 2:** Summary risk estimates of the association between dietary vitamin A and β-carotene intake and the risk of lung cancer.

Subgroups	No.	No.	Risk Estimate (95% CI)	Heterogeneity Test	
	(Cases)	Studies		*I*^2^ (%)	*p*-value
**Vitamin A**	6139	11	0.855 (0.739–0.989)	60.5	0.005
Study design					
Prospective	3258	6	0.869 (0.758–0.980)	52.9	0.060
Case-control	2881	5	0.754 (0.560–1.016)	72.7	0.005
Geographic locations					
America	3353	7	0.915 (0.751–1.114)	61.4	0.016
Asia	1142	3	0.682 (0.556–0.837)	0.0	0.549
Sex					
Males	1981	4	0.697 (0.553–0.879)	38.5	0.181
Females	644	4	0.811 (0.415–1.584)	73.8	0.010
**β-Carotene**	9372	18	0.768 (0.675–0.874)	55.9	0.002
Study design					
Prospective	5395	10	0.867 (0.782–0.962)	6.5	0.382
Case-control	3977	8	0.616 (0.469–0.809)	71.6	0.001
Geographic locations					
America	5593	12	0.742 (0.618–0.890)	59.9	0.004
Europe	2637	3	0.933 (0.814–1.070)	0.0	0.484
Asia	1142	3	0.685 (0.523–0.896)	41.8	0.180
Sex					
Males	2494	4	0.786 (0.612–1.010)	45.0	0.142
Females	2027	7	0.730 (0.549–0.972)	49.0	0.067
Histological type					
Squamous cell carcinoma	1039	6	0.693 (0.480–0.982)	71.2	0.004
Small cell carcinoma	228	3	0.654 (0.416–1.027)	0.0	0.863
Adenocarcinoma	609	6	0.695 (0.452–1.069)	68.1	0.008

### 3.3. β-Carotene and Lung Cancer

**Figure 3:**
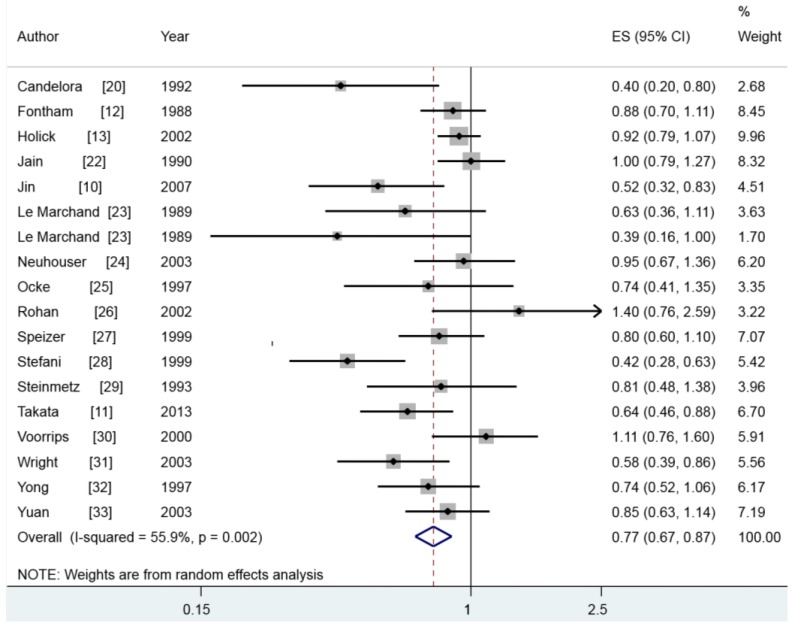
The multivariate-adjusted risk of lung cancer for the highest *versus* lowest categories of β-carotene intake.

Seventeen articles [10,11,12,13,20,22,23,24,25,26,27,28,29,30,31,32,33] with 18 studies involving 9372 lung cancer cases reported the association between dietary β-carotene intake and lung cancer risk. Six of these included studies found a positive relationship between dietary β-carotene intake and lung cancer risk, while 12 studies found a negative result. Pooled results indicated that highest category of β-carotene intake could reduce the lung cancer risk (summary RR = 0.768, 95% CI = 0.675–0.874, *I*^2^ = 55.9%) (Figure 3).

The subgroup analysis by study design was performed, the associations were significant both in prospective studies (RR = 0.867, 95% CI = 0.782–0.962) and in case-control studies (summary RR = 0.616, 95% CI = 0.469–0.809). In subgroup analyses for geographic locations, a positive result was found both in American populations (RR = 0.742, 95% CI = 0.618–0.890) and Asian populations [RR = 0.685, 95% CI = 0.523–0.896], but not in the European populations. Furthermore, when we stratified studies by sex and histological type, the associations were significant only in females and in squamous cell carcinoma. Table 2 showed the detailed results.

### 3.4. Meta-Regression

Moderate of heterogeneities were found both in vitamin A and β-carotene intake and lung cancer risk. Therefore, we used meta-regression with publication years, study design, sex, geographic locations, number of cases and source of controls to explore the potential heterogeneity founded in the analyses. However, the results from meta-regression showed no significant finding in the above-mentioned analyses.

### 3.5. Sensitivity Analysis and Small-Study Effect

Sensitivity analysis showed that the pooled results were not changed while excluded one study at a time. The Egger’s test did not find any significant small-study effect for lung cancer risk with dietary β-carotene intake (*p* = 0.464) or vitamin A intake (*p* = 0.182).

## 4. Discussion

Findings from our study indicated that the highest category of dietary vitamin A intake could reduce the lung cancer risk compared with lowest vitamin A category, especially in prospective studies and Asian populations. The pooled results suggested that highest category of β-carotene intake *versus* lowest category was significantly associated with reduced lung cancer risk. When we performed the subgroup analyses, the associations were significant among American and Asian populations.

Two previous clinical trials had reported that high-dose β-carotene supplementation among smokers and/or asbestos exposed workers could increase the lung cancer risk when compared with the placebo group without any vitamins supplementation [34,35]. The above-mentioned clinical trials assessed β-carotene intake from supplements, indicating that β-carotene from foods or supplements had different effect on lung cancer risk. A possible explanation for dietary β-carotene intake from foods or supplement had different effect on lung cancer risk was due to that the inverse association in the observational studies is an indirect one. Since vegetables and fruit contained many vitamins and minerals, β-carotene is highly correlated with other main carotenoids. The information from specific non carotene carotenoids intake and lung cancer risk is scarce because these carotenoids are available relatively [33]. Another possible explanation for the discrepancy between the epidemiologic literature and intervention studies is the high dose of β-carotene supplement used in the intervention studies than that in the epidemiologic studies that was. This is probably because of the high level used and it acting as a pro-oxidant rather than an antioxidant. In our meta-analysis, we only assess the association between dietary β-carotene intake (not supplements) and lung cancer risk. In the current meta-analysis, we found that a higher category of dietary β-carotene intake (not supplements) could reduce lung cancer risk. The precise mechanisms by which carotenoids might modify lung cancer risk have not been elucidated. However, β-carotene could reduce lung cancer risk by virtue of their provitamin A activity [36], since vitamin A is involved in the control of cell differentiation and proliferation [37]. However, β-carotene might also exert effects independently of their provitamin A activity [36]. In addition, higher category of dietary β-carotene intake resulting from the quenching of singlet oxygen, increased gap junctional intercellular communication, reduced mutagenesis, and enhanced anti-tumor immune responses could reduce the lung cancer risk [38]. In addition to their anti-tumor effects it is possible that some carotenoids might exert procarcinogenic effects.

Vitamin A and β-carotene had been reported associated with some cancers [39,40,41,42]. A recent study had reported that highest category of dietary vegetables and fruits intake could reduce lung cancer risk [43]. β-carotene and vitamins are involved in the vegetables and fruit; this may be a protective effect for lung cancer risk [44]. Antioxidants vitamins included modulation of DNA methylation, repair of DNA damage and induction of detoxifying phase-II enzymes, could prevent the lung cancer [45,46]. In our meta-analysis, we found a significant association for lung cancer risk with higher category of dietary vitamin A and β-carotene intake. This is consistent with the results of the previous meta-analyses about vitamin C and vitamin E.

In our study, significant between-study heterogeneities were found in several analyses. Munafo and Flint [47] had reported that it is common in the meta-analysis about the heterogeneity. A moderate degree of heterogeneity was found in the pooled results and the subgroup analyses. This might have arisen from publication years, study design, sex, geographic locations where the study conducted, cases and source of controls. Therefore, meta-regression and subgroup analyses were performed to explore the potential heterogeneity. However, the *P*-value was all greater than 0.05 for meta-regression analysis and the heterogeneity were presence in some subgroup analyses (Table 2). Lung cancer is a complex etiology and pathophysiology disease. Thus, other unknown confounding factors, such as the possible interaction between genetic and environment variables, may well be a potential cause of the between-study heterogeneity.

Our meta-analysis had some advantages. First, a highlight of our analysis was that higher category of dietary vitamin A and β-carotene intake could significantly reduce lung cancer risk. Second, the current study included many more cases and participants, and this could obtain a more precise result between dietary β-carotene and vitamin A intake and lung cancer risk. Third, small-study effect was not detected in our study.

There were some limitations that should be concerned in our study. First, case-control study may cause the recall or selection bias, and this can improve the potential heterogeneity. Several case-control studies were included in this study. In our results, the association was not significant in case-control studies. However, an inverse association was found between dietary β-carotene intake and lung cancer risk. Although cohort studies can allow a much greater possibility of reaching reasonable conclusions, the case-control study is an important method. Second, the dose-response analysis between dietary β-carotene and vitamin A intake and lung cancer risk was not conducted due to the limited data in each individual study. Further studies with providing dose, cases and person-years for β-carotene and vitamin A are wanted to assess this dose-response association. Third, for the subgroups of geographic locations, significant association was found only among American and Asian populations, but not in the European populations. In addition, only three studies were conducted in Europe. Therefore, the results from our study are more applicable to American and Asian populations. More studies are wanted to assess this association in other countries. Fourth, moderate between-study heterogeneities were found in whole result and some subgroup analyses, but the meta-regression cannot explain the heterogeneity. Thus, other unknown confounders, such as the possible interaction between genetic and environment variables, may be potential contributors to this heterogeneity.

## 5. Conclusions

Findings from this study indicated that dietary vitamin A and β-carotene intake could reduce lung cancer risk. However, dose-response analysis was not performed due to the limited data in each individual article. Due to this limitation, further studies with detailed dose, cases and person-years for each category are wanted to assess this dose-response association.
